# Antiseptics and Disinfectants in Office Practice

**Published:** 1887-07

**Authors:** Alfred T. Peete

**Affiliations:** Branchville, S. C.


ARTICLE II.

### ANTISEPTICS AND DISINFECTANTS IN OFFICE PRACTICE.

BY ALFRED T. PEETE, BRANCHVILLE, S. C.

[Read before the South Carolina Dental Association.]

Every member of this Association is familiar with the chronic controversy respecting the germ theory of disease. That battle has been fiercely contested in the past, and is not yet settled in every detail. But the advocates of that theory have the victory within their grasp to-day, thanks to the labors of distinguished specialists, who have differentiated and described for us so many species of micro-organisms. With reference to those organisms which are found in the oral cavity, we know but little. We cannot state positively whether or not they are an actual cause of disease, and we know next to nothing of their genesis or models of action. Analogy, however, and all the facts at present observed, point to one conclusion: These parasites are neither more nor less than a part of Nature's vast army of scavengers. Their office is to remove whatever is dead and in process of decomposition,—to attack and destroy what is weak, defective, or deficient in vitality—in order that Nature's cycle of death and life, of disorganization and reconstruction, may be continued.

At the present day no one doubts that there are worlds and systems and galaxies beyond the reach of mortal vision—almost beyond the confines of thought, but while recognizing the vastness of creation, in one direction, we sometimes forget that nature's works may be equally without limits in the opposite direction.

Every process of reasoning, and every recorded fact tends to assure us that our microscopes can never reveal more than a small part of the myriads of organisms which flourish and grow wherever there is disease, decay, or death, any more than our telescope can traverse the infinite regions of space.

Respecting most of the parasitic forms which infest the oral cavity, we know, certainly, that where they are present, healthy conditions cannot exist, and that they must be destroyed, or rendered inert, before health can be restored.

Now, I am satisfied that there are, to-day, thousands of dentists who give this subject but little attention, and who regard it as of slight importance. There are many operators, for instance, who daily prepare cavities in teeth, with excavator, bur, and chisel, then (provided there is no apparent bad odor) cleanse such cavities with water alone, dry them and fill at once. Is this practice reasonable or safe? I admit that many teeth are saved by this method, but I believe that many more are lost by the neglect of additional precautions. If we could take account of all the gold fillings inserted, and count the number of final failures, the percentage would be startling. Doubtless a goodly proportion of failures are chargeable to want of thoroughness in excavating or filling; but, remembering the high standard of manipulative skill which obtains, nowadays, and seeing how frequently the work of our finest operators fail, after a few years, I am constrained to believe that there is another and a more potent cause.

How does any operator propose to remove infected matter and disease germs from any cavity? Surely not by means of steel instruments, and a syringe charged with water. Any microscopist would laugh to scorn such an idea. How shall we prevent the *future* entrance of such germs into such a cavity? Not, surely, by the simple insertion of gold, when we are told that a soft gold filling, under a powerful microscope, looks like a bundle of sticks, and a cohesive gold filling appears like a basket of chips; when we know, also, that germs of disease can find an entrance through the cut stem of a peach or apple and infect the whole fruit, we can scarcely rely on mechanical means alone for the preservation of a tooth once attacked by caries. Of some teeth it may be said, as of some men, that "lightning could scarcely kill them" They survive nearly any kind of usage. But, as a rule, I believe that neglect of antiseptic treatment in any case, invites failure. For this reason I never fill the smallest cavity without previous antiseptic treatment; the time required is so little, and the trouble so slight, that neither can be taken into account. Where septic conditions are apparent, I presume no operator would neglect the proper treatment, and I need say nothing on that point.

Let any one who questions the above theory, ask himself why so large a percentage of gold fillings fail in the practice of the most skillful operators, while phosphate of zinc and chloride of zinc fillings (both antiseptic) always preserve teeth, as long as they remain intact in the teeth?

In considering the question of suitable antiseptic agents, in dental practice, we are confronted with a long list, from which every operator must make a selection. He cannot use, or even make fair trial of all. I shall briefly speak of those which have been most effective in my hands.

*Carbolic Acid* is an antiseptic of great power and is somewhat anæsthetic. The objections to its general use are many; it is an irritant poison, has a vile and persistent odor, and requires extreme care in using it. But for one valuable property I would discard it entirely, its value lies in its instantaneous escharotic action, favorable to healthy granulation, hence, it is still the best remedy (in combination) for many forms of ondontalgia. For lining a cavity, after disinfection, and before filling, it is the anti-septic on which I rely. The preparation being—

Tinct. Gum Benzoin, } *a. a.*

Acid Carbol. }

This applied to the dry walls of a cavity forms an impervious antiseptic varnish, while it cauterizes and seals up the canaliculi. It is useful in Stomatitis, etc., but we have other and better remedies in such cases.

*Creosote* I have no use for.

*Iodoform* is slightly disinfectant, and is, possibly, our best antiseptic, because it can be applied in *permanent* form. It is neither irritant nor escharotic, but is strongly anæsthetic. Since it can be given internally in six grain doses, without perceptible effect, it may be considered harmless in practice. Its most striking peculiarity is its healing and cicatrizing power, which makes it the best dressing for a wounded pulp. It is the one *permanent* trustworthy antiseptic for root fillings, and one of the best applications to ulcers and fistulæ. I have over fifty recorded cases of root canals filled with chloro-percha and iodoform, without one failure. These cases included putrid pulps, fungous growth, blind abscess, etc. For preparing sponge graft I now use iodoform, according to Dr. Teague's formula, and find it superior to the old preparation of sponge sterilized with bichloride of mercury. The one objection to this agent is its odor, which is modified by the addition of camphor. If the new preparation, *Iodol*, proves to be equally valuable we can substitute it, and the sole objection will disappear.

*Listerine* is a mild antiseptic, is prepared in elegant form, is harmless and very agreeable, it is a valuable detergent, being an alcoholic preparation, and makes the best of tooth and mouth washes, and gargles. I have cured a fistula, extending from the gum over the right superior lateral, through the alveolus, across the palatal surface to the pharynx, by daily injections of warm water, followed by listerine (full strength) and finally packing the fistulous tract with sponge graft. Listerine is also, peculiarly suited for internal administration. It is not a disinfectant or germicide. Coming to the question of disinfectants and germicides, I know of but one agent that is absolutely free from objections in dental practice, and at the same time, always efficient.

*Bichloride of Mercury*, the most effective germicide, is a deadly poison, and too dangerous for general use.

*Chlorine*, in whatever form, is so powerfully irritant that a variety of evil effects may follow its use.

*Permanganate of Potass.* has the bad property of discoloring every organic substance. I believe that I have seen irritation and inflammation follow from its use in root canals.

*Peroxide of Hydrogen* would be a perfect disinfectant in our practice, if it were not unstable. The extra atom of oxygen is so easily liberated by decomposition that we can never use this agent with entire confidence as to results.

*Sanitas.* In "sanitas" we have the ideal disinfectant, and experiments appear to have sufficiently established its great power as a germicide. It is the most rapid of deodorizers, is harmless, can be given internally, has a pleasant odor, and does not stain clothing. Its great value is shown in the fact that analysis shows a large percentage of peroxide of hydrogen, thymol, eucalyptol, camphoric acid, etc. It has considerable bleaching power.

I use sanitas oil for disinfecting all cavities to be filled, especially root canals. When first penetrating a pulp chamber, I inject sanitas fluid freely, as also into root canals, by means of a bent hypodermic needle, a device shown to me by Dr. Wright. For treatment of ulcers, where an escharotic is not indicated, sanitas gives the best of results. But it has manifold uses. The Sanitas Toilet Fluid is a delightful mouth wash, deodorizing the oral cavity without substituting any other odor. Used in a common atomizer, either of the fluids will remove all odors from the operating room, rendering the atmosphere fragrant as that of the pine woods. They will keep clean and fresh your spittoons, and all vessels about the office. The Sanitas Toilet Soap will remove from the operator's hand and person any suggestion of offensive odors. A little of the fluid should be kept in a glass salt-cellar, in which all instruments should be dipped to cleanse and disinfect them after using. The Sanitas Disinfecting Jelly, resembling vaseline, is an unequalled preparation for dressing wounds, burns and sores. Finally, all these preparations are so cheap that they can be used *ad libitum*. If any member present has not tried them, let me urge him to procure them at once.—*Southern Dental Journal.*

---

ARTICLE III.

